# *Acidovorax citrulli* Type III Effector AopP Suppresses Plant Immunity by Targeting the Watermelon Transcription Factor WRKY6

**DOI:** 10.3389/fpls.2020.579218

**Published:** 2020-11-20

**Authors:** Xiaoxiao Zhang, Yuwen Yang, Mei Zhao, Linlin Yang, Jie Jiang, Ron Walcott, Shanshan Yang, Tingchang Zhao

**Affiliations:** ^1^State Key Laboratory for Biology of Plant Diseases and Insect Pests, Institute of Plant Protection, Chinese Academy of Agricultural Sciences, Beijing, China; ^2^Department of Plant Pathology, University of Georgia, Athens, GA, United States; ^3^Institute of Medicinal Plant Development, Peking Union Medical College, Chinese Academy of Medical Sciences, Beijing, China

**Keywords:** *Acidovorax citrulli*, effector, salicylic acid, watermelon, ClWRKY6, AopP

## Abstract

*Acidovorax citrulli (Ac)* is the causal agent of bacterial fruit blotch (BFB), and BFB poses a threat to global watermelon production. Despite its economic importance, the molecular mechanisms underlying *Ac* pathogenicity and virulence are not well understood, particularly with regard to its type III secreted effectors. We identify a new effector, AopP, in *Ac* and confirm its secretion and translocation. AopP suppresses reactive oxygen species burst and salicylic acid (SA) content and significantly contributes to virulence. Interestingly, AopP interacts with a watermelon transcription factor, ClWRKY6, *in vivo* and *in vitro*. ClWRKY6 shows typical nuclear localization, and AopP and ClWRKY6 co-localize in the nucleus. *Ac* infection, SA, and the pathogen-associated molecular pattern flg22*^*Ac*^* promote ClWRKY6 production, suggesting that ClWRKY6 is involved in plant immunity and SA signaling. Furthermore, ClWRKY6 positively regulates PTI and SA production when expressed in *Nicotiana benthamiana*. Importantly, AopP reduces *ClWRKY6* mRNA and ClWRKY6 protein levels, suggesting that AopP suppresses plant immunity by targeting ClWRKY6. In summary, we identify a novel effector associated with the virulence mechanism of *Ac*, which interacts with the transcription factor of the natural host, watermelon. The findings of this study provide insights into the mechanisms of watermelon immune responses and may facilitate molecular breeding for bacterial fruit blotch resistance.

## Introduction

Bacterial fruit blotch (BFB), caused by the Gram-negative bacterium *Acidovorax citrulli* (*Ac*) (Schaad et al., 1978, 2008; Willems et al., 1992), is a destructive disease that affects cucurbit crops worldwide (Burdman and Walcott, 2012), causing significant economic losses. Perennially, China is the main producing country of watermelon (Xu, 2011). However, the occurrence of BFB disease has severely restricted the healthy development of watermelon production (Zhao, 2012). In recent years, some provinces in China have reported the occurrence of BFB, such as Xinjiang (Zhang and Mo, 1996), Gansu (Xue et al., 2009), and Ningxia (Jin et al., 2014). Although the occurrence of BFB disease has attracted increased attention worldwide, the pathogenic mechanism of *Ac* remains unclear. Similar to most plant pathogenic bacteria, *Ac* uses the type III secretion system (T3SS) to inject type III effector (T3E) proteins into the host to cause disease (Jimenez-Guerrero et al., 2020). However, unlike other well-studied T3SS-dependent pathogenic bacteria, how T3Es perturb innate plant immunity remains mostly unknown for *Ac* (Traore et al., 2019; Jimenez-Guerrero et al., 2020).

The initial stage of plant immunity is typically activated by the recognition of pathogen-associated molecular patterns (PAMPs), leading to a basal immune response referred to as pattern-triggered immunity (PTI) that results in the generation of reactive oxygen species (ROS), callose deposition, and induction of pathogenesis-related gene expression (Zhang et al., 2007; Kang et al., 2014). PAMPs in plant pathogenic bacteria usually include bacterial flagellins, elongation factor Tu (EF-Tu), cold shock proteins, peptidoglycan, and lipopolysaccharide (Boller and Felix, 2009). Currently, detailed investigations are being conducted on the flg22 region of PAMPs. It is a conserved domain near the N terminus of bacterial flagellins, which can trigger PTI, and is recognized by FLS2 in several plant species (Felix et al., 1999). In response to the defense by PTI, phytopathogenic bacteria secrete T3Es directly into host cells to suppress the immune system via interference with gene expression, protein turnover, vesicle trafficking, RNA metabolism, and cellular processes (Block et al., 2008; Block and Alfano, 2011). As a counter-attack strategy, plants, particularly those with higher resistance, exhibit effector-triggered immunity (ETI) to perturb specific T3Es directly or indirectly via cytoplastic, nucleotide-binding, leucine-rich repeat proteins, which are often associated with a burst of localized programmed cell death (Jones and Dangl, 2006; Boller and He, 2009). Our recent study found that a T3E, AopN, in *Ac* induced programmed cell death and triggered the ETI response in *Nicotiana benthamiana* (Zhang et al., 2020). The signaling pathways of PTI and ETI are highly complicated. Plants often activate transcription factors (TFs) to regulate functional genes related to plant immunity in response to PTI or ETI signaling. Several TFs have been found to be related to plant immunity, including MYB, bZIP, NAC, and WRKY; of these, several WRKY TFs have been implicated in the regulation of transcriptional reprogramming associated with plant PTI immune responses (Eulgem and Somssich, 2007). Additionally, plant hormones such as salicylic acid (SA), jasmonic acid, and ethylene are considered to play critical roles in various aspects of the plant immune system (Pieterse et al., 2009, 2012). SA signaling provides resistance against biotrophic and hemibiotrophic pathogens, such as the oomycete *Hyaloperonospora arabidopsidis* and the bacterial pathogen *Pseudomonas syringae*, respectively, whereas jasmonic acid and ethylene mainly contribute to immunity against necrotrophic pathogens such as *Alternaria brassicicola* (Tsuda and Katagiri, 2010). *Arabidopsis* mutants defective in SA biosynthesis or accumulation, such as *ics1*, *eds1*, *pad4*, and *eds5* mutants, showed deficiencies in systemic acquired resistance and enhanced susceptibility to pathogen infection (Lu, 2009). A previous study reported that WRKY TFs, such as AtWRKY70, are involved in SA signaling (Li et al., 2004).

There have been few reports on T3Es in *Ac*, which could be due to the lack of a suitable plant interaction system. We have previously shown that *hrpG* and *hrpX* are the core regulators of T3SS (Zhang et al., 2018), providing a foundation for the screening and identification of T3Es. Recently, a screening system for T3SS inhibitors in *Ac* was established (Ma et al., 2019), which will also be useful for screening of T3E. In addition, some potential candidate T3Es were also screened based on differences in transcription between the *Ac* wild-type (WT) strain M6 and its *hrpX* mutant, as well as by using transcriptome analysis (Jimenez-Guerrero et al., 2020). A recent study confirmed that the model plant *N. benthamiana* could be used to analyze the pathogenic mechanism of *Ac* (Traore et al., 2019). In the past 5–6 years, studies of T3Es in *Ac* have been progressing, from the initial analysis of homologous effector proteins (Eckshtain-Levi et al., 2014) and the possible relationship between effector proteins and host preference (Yan et al., 2017) to the identification of novel T3Es (Jimenez-Guerrero et al., 2020). However, few studies have attempted to identify and determine the function of *Ac* effectors. Recently, we reported that a T3E AopN in *Ac* inhibited the PTI response and induced the ETI response by triggering PCD in *N. benthamiana* (Zhang et al., 2020).

The pathogenic *Ac* strain Aac5 was previously isolated from watermelon (Yan et al., 2013). In the present study, we identified a new effector AopP from the *Ac* strain Aac5 by secretion and translocation analyses. We found that AopP suppressed the PTI pathway, reduced the SA content, and targeted the TF WRKY6 of the natural host watermelon, resulting in plant immunity disruption. More importantly, we found that the deletion of the AopP-encoding gene reduced the pathogenicity of *Ac* strain Aac5 in watermelon, indicating that AopP plays an important pathogenic role.

## Materials and Methods

### Plant Materials and Bacterial Strains

Watermelon plants (cv. Ruixin) were grown at 25°C under a 16 h light cycle, and at 20°C under an 8 h dark cycle at 40–60% relative humidity for bacterial inoculation assays. *N. benthamiana* plants were grown at 25°C under a 16 h light cycle and at 22°C under an 8 h dark cycle at 40–60% relative humidity for transient expression assays. *Escherichia coli* strain DH5α was cultured at 37°C in Luria broth (LB). *Agrobacterium tumefaciens* strain GV3101 was cultured at 28°C in LB for transient expression assays. The *Ac* WT strain Aac5 and its derivative strains were cultured at 28°C in King’s B (KB) and T3SS-inducing broth (Zhang et al., 2018). Additionally, the *Pst* DC3000 *hrcC* mutant, D36E strain, and its derivative strains were cultured at 28°C in KB and *hrp*-inducing medium (Liu et al., 2015). All strains and derived strains are listed in Supplementary Table S1.

### Plasmid Constructs

The promoter sequence of *avrPto1* from the *Pst* DC3000 genome, and its T3SS secretion signal peptide coding sequences containing the first 50 amino acids were synthesized and introduced into pBBRNolac-4FLAG (Zhang et al., 2018) to generate the pBBRavrPto1-4FLAG vector, and then fused with *CyaA* to generate the pBBRavrPto1-CyaA vector. The *CyaA* report gene sequences were also introduced into pBBRNolac-4FLAG to generate the pBBRNolac-CyaA vector. The 3 × FLAG/mCherry/eGFP tag was introduced into the pBI121 vector to generate the pBI121-3 × FLAG/mCherry/eGFP vector for the transient expression assay. All primers used in this assay are listed in Supplementary Table S2.

### Construction of the *aopP* Markerless Mutant

To generate the *aopP* markerless mutant, a 285 bp fragment upstream of the *aopP* open-reading frame and a 364 bp fragment downstream of the *aopP* open-reading frame, were amplified by polymerase chain reaction (PCR) and introduced into the pK18mobsacB vector by triparental mating with an *E. coli* strain carrying the helper plasmid, pRK600. The construct was transformed into the WT Aac5 strain. The markerless mutant screening method was used (Zhang et al., 2018). All primers used in this assay are listed in Supplementary Table S2.

### Effector Identification Assay

The full-length cDNA sequence of *AopP* and its native promoter sequences were cloned and fused with the pBBRNolac-CyaA vector. The construct was then conjugated into the WT strain Aac5 and its type III secretion-defective *hrcJ* mutant by triparental mating. Effector identification assays were performed using western blotting and CyaA translocation reporter assays. The AopP-CyaA protein in cell and supernatant fractions was analyzed as described previously (Liu et al., 2015). Sample proteins were precipitated from the T3SS-inducing medium and cell pellets using 10% trichloroacetic acid and then separated by sodium dodecyl sulfate polyacrylamide gel electrophoresis. Western blot assays were performed using the anti-CyaA antibody (Santa Cruz Biotechnology, Dallas, TX, United States; 1:2500 dilution). The CyaA translocation assay was used to test AopP translocation. Bacterial cells in 10 mM MgCl_2_ were inoculated on the cotyledons of 2-week-old watermelon seedlings at a density of 3 × 10^8^ colony-forming units (CFU)/mL. Leaf disks were collected 8 h after inoculation using a 9-mm-diameter cork-borer. Two disks per leaf, from three different seedlings, made up a sample, for a total of 12 disks per sample. Cyclic AMP (cAMP) levels were detected using a Correlate-EIA cAMP immunoassay kit (Enzo Life Sciences, Farmingdale, NY, United States) according to the manufacturer’s instructions. All primers used in this assay are listed in Supplementary Table S2. Each experiment was independently repeated three times.

### Pathogen Infection Assays

For *Ac* infection assays, qualitative analysis of symptoms was performed using cotyledons (*n* = 6) of 2-week-old watermelon seedlings (cv. Ruixin), which were inoculated with the WT strain Aac5 and *aopP* mutant at a concentration of 1 × 10^4^ CFU/mL using a syringe. Cotyledons were photographed 72 h after inoculation using an EOS 70D camera (Canon, Beijing, China). Quantitative analysis was then performed on inoculated watermelon cotyledons collected 4 and 48 h after inoculation, and bacterial populations were measured and evaluated as described previously (Liu et al., 2015). Each experiment was independently repeated three times.

### ROS Burst Assay

The full-length cDNA sequence of *aopP* was cloned and fused with the pBI121-3 × FLAG vector. Flg22-elicited ROS assays were performed with the transient expression of EV (empty vector, pBI121-3 × FLAG) or AopP using *A. tumefaciens* GV3101 at an optical density at 600 nm (OD_600_) of 0.5. Seven-week-old leaves of *N. benthamiana* were used in this assay. After 36 h, 12 leaf disks (4 mm in diameter) were collected from each inoculated region and were allowed to float in 100 μL of sterile distilled water in a 96-well plate. Next, the water was removed, and a 100 μL solution containing 100 nM flg22, 20 μg/mL horseradish peroxidase, and 100 μM luminol were added to each well. Luminescence was recorded immediately using a Tecan Infinite F200 luminometer (Tecan, Männedorf, Switzerland) for 60 min. Diaminobenzidine (DAB) staining was performed to evaluate differences between watermelon cotyledons inoculated with the WT strain and *aopP* mutant at 1 × 10^8^ CFU/mL using a syringe. After 24 h, the leaves were treated as described previously (Priller et al., 2016; Sang and Macho, 2017).

### Bimolecular Fluorescence Complementation (BiFC) Assay

To generate BiFC constructs, full-length cDNA sequences of *aopP* and *ClWRKY6* were cloned and fused with the pSPYNE^®^173 and pSPYCE(M) vectors, respectively (Waadt et al., 2008). The constructs were co-expressed in *N. benthamiana* leaves for analysis as previously described (Waadt et al., 2008; Luo et al., 2019). After 48 h, the fluorescence signal was visualized under a laser confocal fluorescence microscope (Zeiss LSM 880, Oberkochen, Germany) at an excitation wavelength of 488 nm. Each experiment was independently repeated three times.

### Glutathione S-Transferase (GST) Pull-Down Assay

To generate the expressed constructs, cDNA sequences of *aopP* and *ClWRKY6* were cloned and fused with pGEX6P-1 and pET22b (+) vectors, respectively. Subsequently, AopP-GST and ClWRKY6-His were expressed in *E. coli* strain BL21 and purified. An equal amount of purified ClWRKY6-His was incubated with GST (negative control), or AopP-GST, for 6 h and then passed through a glutathione agarose column. The column was washed five times, and the bound protein was boiled and separated using sodium dodecyl sulfate polyacrylamide gel electrophoresis. Western blotting was performed using anti-GST (MBL, Woburn, MA, United States; 1:5000 dilution) and anti-His antibodies (MBL; 1:5000 dilution). Each experiment was independently repeated three times.

### Luciferase (LUC) Complementation Imaging (LCI) Assay

A firefly LCI assay was performed as previously described (Chen et al., 2008; Yang et al., 2019). AopP-nLUC was inserted into the pCAMBIA-nLUC vector (Chen et al., 2008), and ClWRKY6-cLUC was inserted into the pCAMBIA-cLUC vector (Chen et al., 2008); these constructs were used for LCI assays. *Agrobacterium* GV3101 strains carrying AopP-nLUC and ClWRKY6-cLUC constructs were mixed equally and inoculated into *N. benthamiana* leaves. To examine the interactions between cLUC and nLUC constructs, a CCD imaging apparatus (NightSHADE LB985; Berthold, Bad Wildbad, Germany) was used to measure LUC activity. Each experiment was independently repeated three times.

### Subcellular Localization of AopP and ClWRKY6 and Co-localization Assay

Co-localization was evaluated using AopP::GFP and ClWRKY6::mCherry. AopP fusion with the pBI121-eGFP vector and ClWRKY6 fusion with the pBI121-mCherry vector were generated, and the constructs were transiently expressed in *N. benthamiana* at an OD_600_ of 0.3. After 48 h, the inoculated leaves were visualized under a laser confocal fluorescence microscope (Zeiss LSM 880) at an excitation wavelength of 488 or 588 nm (Chen et al., 2015). After 48 h, the leaves of *N. benthamiana* co-expressing AopP::GFP and ClWRKY6::mCherry were visualized under a laser confocal fluorescence microscope (Zeiss LSM 880) at an excitation wavelength of 488 or 588 nm. Each experiment was independently repeated three times.

### Analysis of mRNA Level

The cotyledons of 2-week-old watermelon seedlings were sprayed with 0.1 μM flg22*^*Ac*^* and 0.1 mM SA until run-off was observed, and total RNA was isolated from the seedlings at different time points using a Quick-RNA Plant Kit (Zymo Research, Irvine, CA, United States; cat. no. R2024). To test watermelon responses to *Ac* infection, the WT strain Aac5 was inoculated onto the cotyledons of 2-week-old watermelon seedlings at 3 × 10^8^ CFU/mL using the spray method, and the total RNA was isolated from the seedlings at different time points using a Quick-RNA Plant Kit (Zymo Research; cat. no. R2024). Quantitative real-time PCR (qPCR) was performed using SYBR Green Real-Time PCR Master Mix (Toyobo, Osaka, Japan). Watermelon *ACTIN* was used as an internal control (Kong et al., 2014). In order to analyze the expression of the PTI marker gene, *Agrobacterium* GV3101 strains showing transient expression of EV (empty vector, pBI121-3 × FLAG), AopP, or ClWRKY6 were inoculated onto *N. benthamiana* leaves. After 36 h, the inoculated leaves were collected and floated in 4995 μL sterile distilled water in a 6-well plate overnight. Next, flg22 was added to 100 nM in each well. Total RNA was isolated from *N. benthamiana* seedlings at different time points using a Quick-RNA Plant Kit (Zymo Research, cat. no. R2024). qPCR was conducted using the SYBR Green Real-Time PCR Master Mix (Toyobo). *N. benthamiana ACTIN* was used as an internal control (Chen et al., 2015). All primers are shown in Supplementary Table S1. Each experiment was independently repeated three times.

### Analysis of Protein Levels

The cotyledons of 2-week-old watermelon seedlings were sprayed with 0.1 μM flg22*^*Ac*^* and 0.1 mM SA until run-off was observed, and the total protein was isolated from leaves collected at different time points using a Plant Protein Extraction Kit (Solarbio, Beijing, China). Protein quantification was performed using a BCA protein kit (Takara, Shiga, Japan). Western blotting was performed using anti-ClWRKY6 antibodies (Gene Universal, Inc., Anhui, China; 1:1000 dilution). To test watermelon responses to *Ac* infection, the WT strain Aac5 was inoculated into the cotyledons of 2-week-old watermelon seedlings at 3 × 10^8^ CFU/mL, and the total protein was isolated from the leaves collected at different time points using a Plant Protein Extraction Kit (Solarbio, Beijing, China). Proteins were quantified using a BCA protein kit (Takara). Western blotting was performed using anti-ClWRKY6 antibodies (Gene Universal, Inc., 1:1000 dilution). Each experiment was independently repeated three times.

### Analysis of D36E Expressing *AopP* Translocation Into Watermelon

Cloning of the AopP 51-642 amino acid coding sequence was performed by introducing the pBBRavrPto1-CyaA vector, and then clicking into the D36E and *hrcC* mutant of *Pst* DC3000 strains. Subsequent detection methods used are described in section “Effector Identification Assay,” except the injected concentration of the strain was 5 × 10^8^ CFU/mL, and it was sampled after 12 h. The *avrPto1* native promoter and its T3SS secretion signal peptide sequences and coding sequences containing the first 50 amino acids was a synthesis-based *Pst* DC3000 genome according to the KEGG database. Each experiment was independently repeated three times.

### Quantification of SA Content

Salicylic acid content was quantified as previously described (Stork et al., 2015). Sample preparation prior to SA quantification in watermelon plants differed slightly. First, we constructed the D36E strain expressing *aopP*. The primers used are listed in Supplementary Table S2. Then, we fused AopP (containing 51–462 amino acids) to the above vectors and transferred it into D36E by clicking with 1.8 KV. An empty vector was transferred into D36E as a control. For watermelon plants, the D36E strain carrying *aopP* and an empty vector (EV) was inoculated into the cotyledons of 2-week-old watermelon seedlings at 5 × 10^8^ CFU/mL, and the leaves were collected 24 h after inoculation. Two cotyledons were collected for each seedling, with a total of six cotyledons per sample and six technical replicates per treatment. The SA content of the samples was determined by high-performance liquid chromatography. The method used to determine the effect of ClWRKY6 on the SA content of *N. benthamiana* was similar to that described above (Stork et al., 2015) with the exception that ClWRKY6 fused with mCherry was transiently expressed in *N. benthamiana*, and the leaves were collected 48 h later to detect the SA content. A negative control was set up in which the mCherry expressed in *N. benthamiana* was determined. Each experiment was independently repeated three times.

### Analysis of Whether ClWRKY6 Is Involved in PTI Response

ClWRKY6::mCherry and mCherry were transiently expressed in *N. benthamiana* at an OD_600_ of 0.5 each. After 24 h, the D36E strain at 5 × 10^8^ CFU/mL concentration was injected into the *N. benthamiana* leaves that were transiently expressed with ClWRKY6::mCherry and mCherry. Four days later, a hole punch was used to collect leaf samples of approximately 12 mm, and then the number of colonies was counted.

### Analysis of the Effect of AopP on the Expression of *ClWRKY6*

For the analysis of transcription levels, the D36E expressing AopP was injected into the cotyledons of 2-week-old watermelon seedlings at a concentration of 5 × 10^8^ CFU/mL. A control sample was collected immediately (at 0 h). Then, samples were collected at 3 and 6 h. The RNA of the samples was extracted and reverse transcribed into cDNA. The qPCR was performed using SYBR Green Real-Time PCR Master Mix (Toyobo, Osaka, Japan). Watermelon *ACTIN* was used as an internal control (Kong et al., 2014). Each experiment was independently repeated three times.

To analyze the protein co-expression levels of AopP fused with FLAG and ClWRKY fused with HA in *N. benthamiana*, leaves were collected 2 days after agroinfiltration. The co-expression levels of mCherry fused with FLAG and of ClWRKY fused with HA in *N. benthamiana* leaves collected 2 days after agroinfiltration were used as positive controls. After extracting the total protein for quantification, western blotting was performed to analyze the difference in protein expression.

### Statistical Analysis

Data were analyzed by one-way analysis of variance and Tukey’s honest significant difference tests. For qPCR, data were analyzed by independent-sample *t*-tests. Statistical analyses were conducted using SPSS version 17.0 (SPSS, Inc., Chicago, IL, United States) and GraphPad PRISM 5.0 software (GraphPad, Inc., La Jolla, CA, United States). Differences in results with *p*-values < 0.05 were considered significant.

## Results

### AopP Is a T3E Protein of *Ac*

**FIGURE 1:**
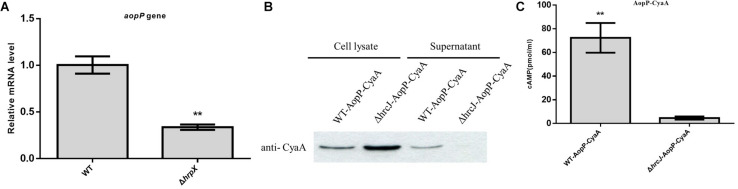
Identification and analysis of AopP as a type III effector. **(A)** Analysis of transcription differences of *AopP* in the wild-type (WT) Aac5 strain and *hrpX* mutant. The WT Aac5 and *hrpX* mutant strains were cultured in King’s B medium to the logarithmic growth phase, then centrifuged at 5000 × *g* for 2 min to collect bacterial cells, resuspended in T3SS-inducing medium to OD_600_ = 0.5, induced to extract RNA 3 h after incubation, reverse transcribed into cDNA, and detected by qPCR. Asterisks above bars indicate significant differences as determined by *t*-test, *p* < 0.05. **(B)** Identification and analysis of AopP as a type III effector by western blotting. The wild-type (WT) strain and T3SS-deficient *hrcJ* mutant strain expressing AopP-CyaA fusion protein cultivated in King’s B medium to the logarithmic growth phase in T3SS induction medium for 4 h, following extraction of intracellular components and exocrine protein components using anti-CyaA antibody detection, in which the signal was detected in the intracellular component, but no signal was detected in the *hrcJ* mutant carrying the AopP-CyaA exocrine component. **(C)** Identification and analysis of AopP as a type III effector by the enzyme-linked immunosorbent assay. The WT strain and T3SS-deficient *hrcJ* mutant carrying AopP-CyaA fusion protein were cultivated to the logarithmic phase, resuspended with 10 mM MgCl_2_ at 1 × 10^8^ CFU/mL, and then injected into watermelon leaves. After 8 h, they were sampled with a puncher (diameter: 9 mm) and then analyzed using a correlate-EIA cAMP immunoassay. Asterisks above bars indicate significant differences as determined by *t*-test, *p* < 0.05.

The transcriptional level of *AopP* was reduced in the *hrpX* mutant compared to that in the WT Aac5 strain cultured under T3SS-inducing medium conditions (Figure 1A). The *AopP* gene encodes 642 amino acids. BLAST comparison analysis found that AopP has homology with XopP from *Xanthomonas oryzae* pv. *oryzae* (MAFF 311018), a previously reported effector (Ishikawa et al., 2014). Bioinformatics analysis showed that the protein sequence of AopP had 17% similarity with XopP (*Xoo3222*) (Supplementary Figure S1). To confirm that AopP was a T3E, secretion and translocation were performed by western blotting and CyaA translocation reporter assays. The results demonstrated that AopP-CyaA was present in the cell lysates of the WT Aac5 strain and its Δ*hrcJ* mutant; however, no signal was detected in the supernatants of the Δ*hrcJ* mutant (Figure 1B). These findings showed that AopP was secreted from cells depending on the T3SS activity. To further test whether AopP can translocate into plant cells, we inoculated the strains harboring AopP-CyaA into watermelon leaves and analyzed the cAMP levels, a product of the CyaA-catalyzed reaction, 8 h after inoculation. Natural host watermelon tissue inoculated with WT Aac5 (expressing AopP-CyaA) produced high levels of cAMP. However, the Δ*hrcJ* mutant expressing AopP-CyaA showed significantly lower levels of cAMP in leaves than in leaves inoculated with the WT Aac5 (expressing AopP-CyaA) (Figure 1C). These results confirmed that AopP was indeed a T3E in *Ac.*

### AopP Contributed to Virulence in Watermelon Plants

**FIGURE 2:**
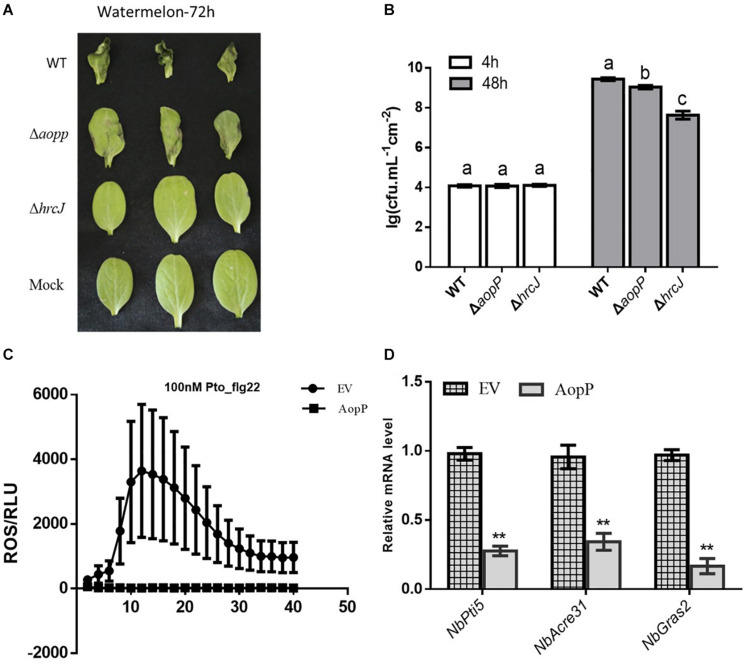
AopP contributing to virulence in watermelon plants and inhibition of pattern-triggered immunity (PTI) responses. **(A)** Qualitative virulence analysis of AopP. The WT Aac5 strain and *AopP* mutant were cultivated to the logarithmic phase, resuspended with 10 mM MgCl_2_ at 1 × 10^4^ CFU/mL, and then injected into the watermelon leaves. Photos were acquired after 72 h to record symptoms. **(B)** Quantitative virulence analysis of AopP. The WT Aac5 strain and *AopP* mutant were cultivated to the logarithmic phase, resuspended with 10 mM MgCl_2_ at 1 × 10^4^ CFU/mL, and then injected into the watermelon leaves. Samples were collected at 4 and 48 h after injection, and bacterial growth was measured and statistically analyzed. Different letters above bars indicate statistically significant differences as determined by one-way analysis of variance and Tukey’s honest significant difference test, *p* < 0.05. **(C)** AopP inhibiting PAMP flg22-induced reactive oxygen species (ROS) burst in *Nicotiana benthamiana. Agrobacterium tumefaciens* strain GV3101 carrying AopP-GFP suppressed ROS production induced by flg22 in *N. benthamiana* unlike that carrying GFP controls. **(D)** Analysis of the effect of AopP on pattern-triggered immunity (PTI). The PTI marker genes *NbPti5*, *NbAcre31*, and *NbGras2* were detected by expressing AopP in *N. benthamiana*. GV3101 carrying an empty vector was used as a control. Asterisks above bars indicate significant differences as determined by *t*-test, *p* < 0.05.

Watermelon leaves inoculated with Δ*AopP* strains exhibited reduced symptom development compared with those inoculated with the WT strain (Figure 2A). Furthermore, bacterial growth analysis revealed that the Δ*AopP* strains had reduced population levels compared with the WT strain (Figure 2B). These results showed that AopP contributed to the virulence of *Ac* in watermelon plants.

### AopP Is Able to Suppress PTI in *N. benthamiana*

Transient expression of AopP in *N. benthamiana* significantly suppressed flg22-triggered ROS burst compared to that in the EV control (Figure 2C) GV3101, carrying the empty vector (pBI121-3 × FLAG). In previous studies, *NbPti5*, *NbAcre31*, and *NbGras2* in *N. benthamiana* were commonly used to determine the level of the PTI response (Nguyen et al., 2010; Chen et al., 2015). We further analyzed whether AopP inhibited PTI marker genes. As shown in Figure 2D, the expression levels of *NbPti5*, *NbAcre31*, and *NbGras2* were significantly reduced with the AopP treatment compared to that with the EV control treatment after flg22 treatment. These results indicate that AopP suppressed plant PTI responses. To explore whether AopP can suppress natural host watermelon PTI responses, the WT strain Aac5 and Δ*AopP* mutant were inoculated into watermelon leaves at 3 × 10^8^ CFU/mL. DAB staining showed that the Δ*AopP* mutant induced higher ROS production levels than the WT Aac5 strain and the mock control using qualitative methods (Supplementary Figure S2). The results also showed that the effector AopP suppressed plant PTI responses by interfering with ROS burst.

### AopP Physically Interacts With ClWRKY6

**FIGURE 3:**
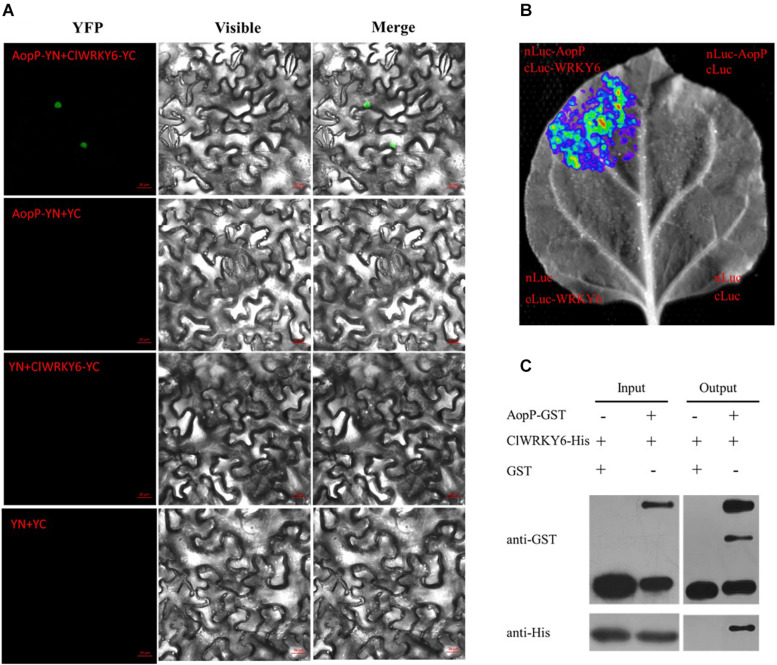
Interaction between AopP and ClWRKY6. **(A)** AopP interacted with watermelon ClWRKY6 as shown by the BiFC assay. AopP fused with nYFP, and ClWRKY6 fused with cYFP constructs were introduced into GV3101 strains. GV3101 carrying 35S::AopP-nYFP or 35S::ClWRKY6-cYFP were co-injected into *Nicotiana benthamiana* leaves. After 48 h, leaves were collected and observed using a confocal microscope. The empty vector nYFP or cYFP was used as a negative control. **(B)** AopP interacted with watermelon ClWRKY6 as observed with the LCI assay. AopP fused with nLUC and ClWRKY6 fused with cLUC constructs were introduced into GV3101 strains. The GV3101 carrying 35S::AopP-nLUC or 35S::ClWRKY6-cLUC were co-injected into *N. benthamiana* leaves. After 48 h, the leaves were collected and observed using a CCD imaging apparatus (NightSHADE LB985; Berthold). **(C)** AopP interacted with watermelon ClWRKY6 as indicated by the GST pull-down assay. The AopP was co-purified with ClWRKY6. AopP-GST or GST was incubated with ClWRKY6-His and precipitated by glutathione agarose. The presence of ClWRKY6-His in glutathione agarose-bound protein was detected by anti-His immunoblot. The experiment was independently repeated three times and similar results were obtained.

**FIGURE 4:**
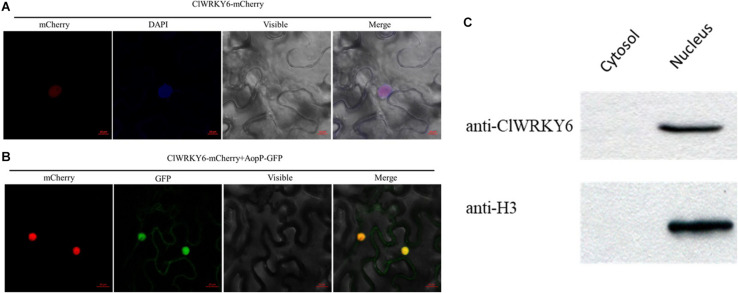
Subcellular localization of ClWRKY6. **(A)** ClWRKY6 localization in the nucleus of *Nicotiana benthamiana* cells. The leaves injected with the *Agrobacterium tumefaciens* GV3101 strain carrying 35S::ClWRKY6-GFP were sampled and observed using a confocal microscope (20×). DAPI staining was used as a control for nuclear localization. **(B)** Co-localization observation of ClWRKY6 and AopP. The leaves were injected with *A. tumefaciens* GV3101 strain carrying 35S::ClWRKY6-mCherry and 35S::AopP-GFP at a ratio of 1:1 and then observed using a confocal microscope (20×). **(C)** Analysis of ClWRKY6 localization in watermelon. The cytoplasmic and nuclear components in watermelon leaves were extracted, and an anti-ClWRKY6 antibody was used to detect signals. The H3 antibody was used as a positive control for nuclear components.

To determine the molecular mechanisms underlying the virulence function of AopP, we used an *Ac*-induced watermelon cDNA library to screen for AopP interactors via yeast two-hybrid (Y2H) assays. A putative WRKY family protein encoded by Cla97C10G206240 from watermelon genome 97103, named WRKY6, was found to interact with AopP (Supplementary Figure S3). These findings suggest that AopP might target signaling components upstream of the SA pathway. BLAST analysis showed that ClWRKY6 had 27% homology with *Arabidopsis* WRKY70 (Supplementary Figure S4). WRKY70 has been found to regulate the SA pathway in *Arabidopsis* (Li et al., 2004). Therefore, we hypothesized that AopP might interact with ClWRKY6. To test this hypothesis, the interaction between AopP and WRKY6 was analyzed by BiFC, LCI, and GST pull-down assays. BiFC assays were performed to test the interaction between AopP and WRKY6 in *N. benthamiana*. As shown in Figure 3A, co-expression of AopP fused with nYFP and of WRKY fused with cYFP in *N. benthamiana* leaves 2 days after agroinfiltration, showed fluorescent signals in the nucleus of infiltrated cells (first panel of Figure 3A). However, co-expression of AopP-nYFP and cYFP, nYFP and WRKY6-cYFP, and nYFP and cYFP in cells of *N. benthamiana* leaves 2 days after agroinfiltration did not show a fluorescent signal. These results confirmed that AopP interacts with WRKY6 *in vivo.* We further analyzed the *in vitro* interaction between AopP and WRKY6 by the GST pull-down assay. As shown in Figure 3C, GST-AopP or GST was incubated with polyhistidine (His)-tagged ClWRKY6, precipitated with glutathione agarose, and tested for the presence of ClWRKY6. GST-AopP, but not GST alone, co-purified with His-tagged ClWRKY6 (Figure 3C), indicating a direct interaction between AopP and ClWRKY6. To qualitatively detect the interaction between AopP and ClWRKY6, an LCI assay was performed. As shown in Figure 3B, co-expression of AopP-nLUC and ClWRKY6-cLUC in *N. benthamiana* leaves 2 days after agroinfiltration showed a chemiluminescence signal, whereas the negative control treatment did not, indicating a direct interaction between AopP and ClWRKY6. These results indicate that AopP directly targets and interacts with ClWRKY6 *in vivo* and *in vitro*. Further experiments were performed to clarify protein localization, subcellular localization, and co-localization. After 48 h of transient expression of 35S::ClWRKY6-mCherry in *N. benthamiana* leaves by *Agrobacterium*-mediated transient transformation, ClWRKY6-mCherry fluorescence was observed exclusively in the nucleus (Figure 4A). To confirm ClWRKY6 localization in watermelon plants, we extracted cytoplasmic and nuclear fractions from watermelon cells. Western blotting with anti-ClWRKY6 antibodies revealed a signal with a nuclear component similar to anti-H3 (positive control); however, no signals were observed in the cytoplasm (Figure 4C), indicating the presence of ClWRKY6 in the nucleus. After 48 h of introducing 35S::ClWRKY6-mCherry and 35S::AopP-GFP into *N. benthamiana* leaves, superimposed yellow fluorescence was observed exclusively in the nucleus (Figure 4B), revealing the co-localization of AopP and ClWRKY6 in the nucleus of plants.

### AopP Reduces SA Production in Watermelon

**FIGURE 5:**
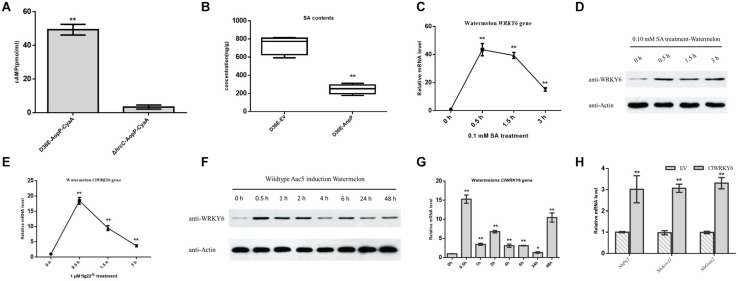
Effect of AopP on the salicylic acid (SA) content and involvement of ClWRKY6 in the immune response and salicylic acid (SA) signaling. **(A)** Identification and analysis of AopP as a type III effector by the enzyme-linked immunosorbent assay. The D36E strain and *hrcC* mutant of *Pst* DC3000 carrying AopP-CyaA fusion protein were cultivated to the logarithmic phase, resuspended with 10 mM MgCl_2_ at 5 × 10^8^ CFU/mL, and then injected into watermelon leaves. After 12 h, they were sampled with a puncher (diameter 9 mm) and then analyzed using a correlate-EIA cAMP immunoassay. **(B)** Watermelon leaves were injected with the D36E strain expressing *aopP* or empty vector (negative control) at 5 × 10^8^ CFU/mL (resuspended in 10 mM MgCl_2_). After 24 h, the leaves were collected and SA was quantitatively detected by high-performance liquid chromatography. **(C–E)** Expression patterns of *ClWRKY6* in watermelon at the indicated time points after treatment with SA **(C,D)**, flg22 **(E)**, and wild-type (WT) Aac5 strain **(F,G)**. **(D,F)** The protein level of ClWRKY6 in watermelon at the indicated time points after treatment with SA and WT Aac5 strain. The antibody ClWRKY6 was used in the assay, and the plant Actin antibody was used as a control. **(H)** Analysis of the effect of ClWRKY6 on pattern-triggered immunity (PTI). The PTI marker genes *NbPti5*, *NbAcre31*, and *NbGras2* were detected by expressing ClWRKY6 in *Nicotiana benthamiana*. GV3101 carrying an empty vector was used as a control. Asterisks above bars indicate significant differences as determined by *t*-test, *p* < 0.05.

Natural host watermelon tissue inoculated with D36E (expressing AopP-CyaA) produced high levels of cAMP. However, the Δ*hrcC* mutant, *Pst* DC3000 T3SS-deficient strain without T3E secretion function and expressing AopP-CyaA, had significantly lower levels of cAMP in the leaves than leaves inoculated with D36E (expressing AopP-CyaA) (Figure 5A). These results indicate that AopP can translocate into watermelon cells via the D36E expression system. Based on this, we constructed the FLAG fusion reporter strain to reduce the effect of CyaA on protein function. The D36E strain carrying AopP fused with FLAG was inoculated into watermelon leaves, and the D36E strain carrying an empty vector (pBBRavrPto1-4 × FLAG) was used as a positive control. After 24 h, high-performance liquid chromatography results revealed that leaves inoculated with the D36E strain carrying AopP had significantly reduced the SA content compared to the positive controls (Figure 5B), indicating that AopP suppressed SA signaling in watermelon plants.

### ClWRKY6 Was Involved in the Immune Responses and SA Signaling in Plants

Salicylic acid treatment rapidly induced *ClWRKY6* expression (Figure 5C), and western blot analysis showed that ClWRKY6 was induced by SA treatment (Figure 5D), suggesting that ClWRKY6 is involved in SA signaling. To evaluate the potential role of ClWRKY6 in plant immunity, we investigated whether *ClWRKY6* expression was induced after the PAMP treatment (flg22*^*Ac*^*) from FliC (ABM34940). The flg22*^*Ac*^* peptide sequence is the same as flg22*^*Aa*^* and flg22*^*Xcc*^*, as previously reported (Wang et al., 2015). qPCR results showed that *ClWRKY6* expression rapidly increased after flg22*^*Ac*^* treatment (Figure 5E). We further analyzed whether *ClWRKY6* expression was induced during infection by the WT strain using qPCR and western blotting. Notably, infection with the WT strain significantly induced *ClWRKY6* mRNA and ClWRKY6 protein levels (Figures 5F,G). Importantly, the qPCR analysis also showed that *ClWRKY6* expression in *N. benthamiana* enhanced the expression of PTI marker genes, *NbPti5*, *NbAcre31*, and *NbGras2*, compared to that in the EV control (Figure 5H). These results indicate that ClWRKY6 is involved in SA signaling and participates in immune responses to *Ac* infection.

### AopP Is Able to Suppress the Expression of *ClWRKY6*

**FIGURE 6:**
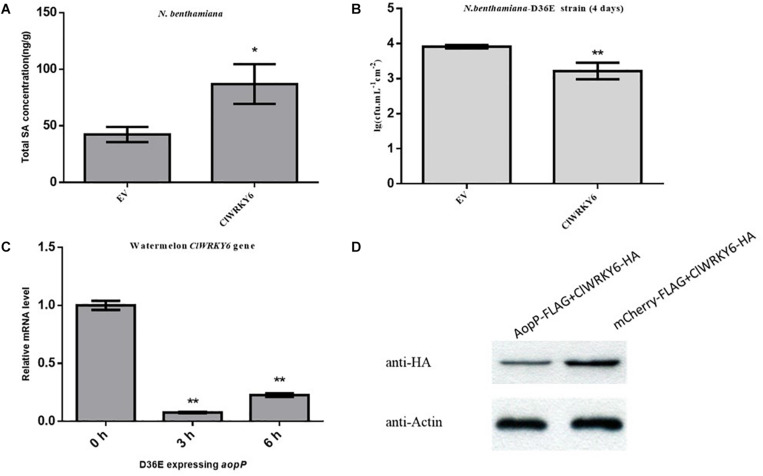
Effect of AopP on the expression of *ClWRKY6*. **(A)** *Nicotiana benthamiana* leaves were injected with the GV3101 strain expressing AopP or empty vector (negative control) at an optical density at 600 nm (OD_600_) of 0.5. After 48 h, the leaves were collected, and SA was quantitatively detected by high-performance liquid chromatography. **(B)** Analysis of whether ClWRKY6 is involved in PTI response. **(C)** Analysis of the effect of the AopP transcription level on ClWRKY6. **(D)** Analysis of the effect of the AopP protein level on ClWRKY6. Asterisks above bars indicate significant differences as determined by *t*-test, *p* < 0.05.

High-performance liquid chromatography results revealed that leaves inoculated with the GV3101 strain carrying ClWRKY6 had significantly higher SA content compared with the negative controls (Figure 6A), suggesting that ClWRKY6 positively regulates SA signaling when expressed in *N. benthamiana.* Past studies have shown that the D36E strain is involved in the PTI pathway in *N. benthamiana* (Wei et al., 2018). When ClWRKY6-mCherry was transiently expressed in *N. benthamiana*, the D36E strain was inoculated into *N. benthamiana* leaves after 24 h. Four days later, the leaves inoculated with the GV3101 strain carrying ClWRKY6-mCherry showed a significantly reduced D36E growth ability compared with the leaves inoculated with the GV3101 strain carrying mCherry (Figure 6B), suggesting that ClWRKY6 positively regulates PTI when expressed in *N. benthamiana.* Based on this, we analyzed the effect of AopP on ClWRKY6 transcription and protein levels. Notably, infection with the D36E strain expressing *aopP* significantly induced *ClWRKY6* mRNA at 3 and 6 h compared to 0 h in watermelon, indicating that AopP suppresses the mRNA expression of *ClWRKY6* (Figure 6C). Importantly, co-expression of AopP fused with FLAG and of ClWRKY fused with HA in *N. benthamiana* leaves 2 days after agroinfiltration showed that the expression of *ClWRKY6* was inhibited compared with the co-expression of mCherry fused with FLAG and of ClWRKY6 fused with HA in *N. benthamiana* leaves (negative control) when the internal reference protein expression was consistent. This indicates that AopP is able to suppress the expression of *ClWRKY6* (Figure 6D).

## Discussion

Bacterial fruit blotch caused by *Ac* is a severe seed-borne disease of cucurbits worldwide, resulting in significant economic losses (Burdman and Walcott, 2012). However, few studies have evaluated the pathogenic mechanisms of *Ac*, particularly the key virulence functions of effector proteins. In the past 2 years, research on effectors in *Ac* has progressed substantially. For example, the interaction system between *Ac* and the model plant *N. benthamiana* was established (Traore et al., 2019), and the use of transcriptome technology to screen putative T3Es was reported (Jimenez-Guerrero et al., 2020). However, the functional mechanisms of *Ac* effectors in watermelon plants remain unclear. Accordingly, in the present study, we evaluated the virulence mechanisms of effector AopP in *Ac.*

A previous study showed that *hrpX* is a key regulator of T3SS in the Aac5 strain (Zhang et al., 2018). Additionally, the *hrpX* mutant of the Aac5 strain showed a loss of virulence in natural host watermelon plants (Zhang et al., 2018). Moreover, in the *Ac* M6 strain, *hrpX* has also been shown to regulate some T3Es (Jimenez-Guerrero et al., 2020). Thus, it is feasible to use the *hrpX* mutant to assess the roles of effectors. In the present study, we determined the roles of a newly identified *Ac* effector, AopP (the coding gene is 100% homologous to *Aave_0588* from the AAC00-1 genome, GenBank accession NC_008752), in virulence and plant immunity, including suppression of ROS production and SA signaling, in watermelon and *N. benthamiana*. Importantly, we demonstrated that AopP interacted with the natural host watermelon TF ClWRKY6, which is related to plant immunity and SA signaling. To the best of our knowledge, the present study is the first to analyze the immune effects of *Ac* effectors in watermelon plants as a natural host.

It is critical to screen for relevant effector proteins as molecular probes to explore the pathogenic function of microbes within their natural host. Research on effectors in *P. syringae* (Xin et al., 2016; Wei et al., 2018) and *Xanthomonas* spp. (Song and Yang, 2010; Qin et al., 2018) has been extensively conducted. However, the screening and identification of key effectors in *Ac* remain challenging. We first confirmed the secretion and translocation of the effector AopP using western blotting and a CyaA reporter system, which are commonly used for T3Es (Wang et al., 2007; Block et al., 2010). There have been major advancements in the technology for identifying *P. syringae* effectors; thus, more than 60 effector candidates have been analyzed using ΔAvrRpt2 reporter fusion with the native promoter, which induced programmed cell death in *Arabidopsis thaliana* (Chang et al., 2005; Vinatzer et al., 2005). Additionally, improved methods for fusing constitutive promoters with the ΔAvrRpt2 reporter have been developed (Macho et al., 2009). However, we performed western blotting and CyaA reporter assays rather than using the ΔAvrRpt2 reporter system in *Ac* because it is unclear whether *A. thaliana* can induce the T3SS expression of *Ac.* We confirmed that the natural host, watermelon, stimulated the T3SS of *Ac*, and T3SS defects resulted in a loss of pathogenicity in watermelons (Johnson et al., 2011; Zhang et al., 2018). A recent study also showed that a new T3SS secretion reporter containing the β-lactamase gene fused with a signal peptide sequence of the T3SS effector gene of *Ac* could be used to screen for T3SS inhibitors using a high-throughput screening system (Ma et al., 2019). This system may also be used to identify effectors.

Based on the identification of the effector AopP, we analyzed the effects of AopP on ROS, SA content, and virulence in watermelon, which are important biological functions for determining the roles of AopP as an effector during plant infection. The results of our study showed that AopP inhibited ROS burst, reduced the SA content, and contributed to virulence in watermelon. Importantly, we used the D36E strain (*Pseudomonas syringae* pv. *tomato* DC3000-derived strain) (Wei et al., 2018) to express the *Ac* effector to analyze the effects of AopP on the natural host (watermelon), in which genetic manipulation is difficult and the interaction with *Ac* effectors is challenging to evaluate within a short time using transgenic technology. A previous study demonstrated that the D36E strain only induced the PTI pathway because of the presence of flagella and the T3SS genes. However, the deletion of 36 known effectors (Wei et al., 2015) indicated that this strain was a good natural effector expression system and might be used to overcome limitations with the natural host, proving that D36E-expressed AopP can be secreted into watermelon cells (Figure 5A). By analyzing the natural host using the D36E strain expressing the AopP effector of *Ac* and combining the results of our findings regarding the interplay of *N. benthamiana* and *Ac*, we can better analyze the functions of *Ac* effectors in future research.

A previous study showed that the AopP homologous protein XopP interacted with OsPUB44 in rice (Ishikawa et al., 2014). To identify the molecular target of AopP in watermelon, we screened an *Ac*-induced watermelon cDNA library for plant immunity-related molecular targets interacting with AopP. We found that ClWRKY6, a putative TF in watermelon, interacted with AopP. Further *in vivo* and *in vitro* interaction analyses verified the direct interaction between AopP and ClWRKY6. This is the first report demonstrating that the *Ac* effector AopP targeted its natural host, watermelon, but we did not screen the homologous protein of OsPUB44 in watermelon. It also indicates that AopP may be functionally different from XopP. A previous study showed that the ClWRKY6 homologous protein AtWRKY70 was involved in the SA pathway (Li et al., 2004). In the present study, we found that AopP inhibited the SA content in watermelon; thus, we hypothesized that an interaction between AopP and ClWRKY6 interfered with SA signaling in watermelon. Additional experiments confirmed that ClWRKY6 is involved in SA signaling and showed the typical nuclear localization of a TF. We also confirmed that AopP inhibited the expression of *ClWRKY6* in watermelon (based on mRNA levels) and *N. benthamiana* (based on the protein level). Importantly, ClWRKY6 positively regulated PTI and SA signaling in *N. benthamiana*. SA signaling is involved in PTI pathways. However, immune signaling pathways in watermelons have not been extensively studied, and the use of effector proteins as molecular probes is required to provide additional insights into the mechanisms of watermelon immune signaling pathways. Despite recent advancements in watermelon CRISPR technology (Tian et al., 2017), the applications of this technology remain limited. SA-mediated resistance is critical, and some phytopathogenic bacteria deploy effector proteins to disrupt SA signaling. *Xanthomonas campestris* T3E XopJ targets the host cell proteasome to suppress SA-mediated plant defense (Ustun et al., 2013), and *P. syringae* T3E HopM1 suppresses SA-dependent plant immunity by interacting with min7 (Debroy et al., 2004). The T3E HopI1-containing J domain from *P. syringae* suppresses SA-dependent defenses (Jelenska et al., 2007). A recent study showed that the effector AvrPto1B from *P. syringae* disrupts SA signaling by interacting with NPR1, a key regulator of SA signaling (Chen et al., 2017). However, only a few studies have evaluated SA-mediated resistance in watermelon. Recently, SA-mediated immunity was reported to be involved in resistance against nematodes in watermelon (Yang et al., 2018); however, the effect of SA on watermelon responses to *Ac* infection remains unclear. To the best of our knowledge, the present study is the first to reveal that an effector protein in *Ac* inhibits the SA content of watermelon and interacts with a TF, ClWRKY6, which is closely associated with SA signaling.

## Conclusion

We observed that the *Ac* effector AopP inhibited the PTI pathway by suppressing ROS production and reduced SA content in watermelon. Moreover, AopP contributed to virulence and interacted with a TF, ClWRKY6, in watermelon. ClWRKY6 is involved in plant immunity, and enhanced PTI and SA content when expressed in *N. benthamiana*. Importantly, we described a novel virulence mechanism of *Ac* by modulating the expression of *ClWRKY6* at the mRNA and protein levels. AopP modulated SA signaling to inhibit plant immunity by interacting with ClWRKY6. This is the first report showing that *Ac* suppressed immunity in its natural host watermelon by targeting a TF. The findings of this study provide important insights into the mechanisms of watermelon immune responses and may facilitate studies of molecular breeding with regard to resistance against bacterial fruit blotch.

## Data Availability Statement

The original contributions presented in the study are included in the article/Supplementary Material, further inquiries can be directed to the corresponding author.

## Author Contributions

TZ and XZ planned and designed the research. XZ, YY, LY, JJ, and SY performed the experiments. XZ, YY, and SY analyzed the data. XZ, MZ, and RW wrote the manuscript. All authors contributed to the article and approved the submitted version.

## Conflict of Interest

The authors declare that the research was conducted in the absence of any commercial or financial relationships that could be construed as a potential conflict of interest.

## Supplementary Material

Supplementary Figure 1 AopP homology analysis. Analysis of amino acid homology of AopP with XopP (*Xoo3222*) (Ishikawa et al., 2014).

Supplementary Figure 2 DAB staining for detecting ROS with WT and the *AopP* mutant in watermelon. DAB staining analysis of ROS burst in watermelon leaves. The WT Aac5 strain and *AopP* mutant were cultivated to a logarithmic phase, resuspended with 10 mM MgCl_2_ at 1 × 10^8^ CFU/mL, and then injected into the watermelon leaves. After 24 h, the leaves were stained with DAB solution. After staining, images were captured with a camera.

Supplementary Figure 3 Yeast two-hybrid with AopP and ClWRKY6. Cloning the *AopP* and *ClWRKY6* coding sequences inserted into pGBKT7 and pGADT7 vectors, respectively. Subsequent co-transformation of the Gold2 strain and set positive control (co-transformation of pGBKT7-53 and pGADT7-T into Gold2 strain) and negative control (co-transformation of pGBKT7-lam and pGADT7-T into Gold2 strain). The Gold2 yeast strain was transformed with the above constructs, and the yeast assay was performed as described previously (Yang et al., 2014). Each experiment was independently repeated three times.

Supplementary Figure 4 ClWRKY6 homology analysis. Analysis of amino acid homology of ClWRKY6 with WRKY70 (Uniport: Q9LY00). The ClWRKY6 coding sequence is gene ID Cla97C10G206240 according to 97103 Watermelon V2 genome.

Supplementary Table 1 Bacterial strains and plasmids used in this study.

Supplementary Table 2 List of all the primer sequences used in this study.
