# Supplementary figures and images for: Acidovorax citrulli Type III Effector AopP Suppresses Plant Immunity by Targeting the Watermelon Transcription Factor WRKY6
Source: Front Plant Sci. 2020 Nov 20;11:579218. doi: 10.3389/fpls.2020.579218 (PMC7718035; doi:10.3389/fpls.2020.579218)

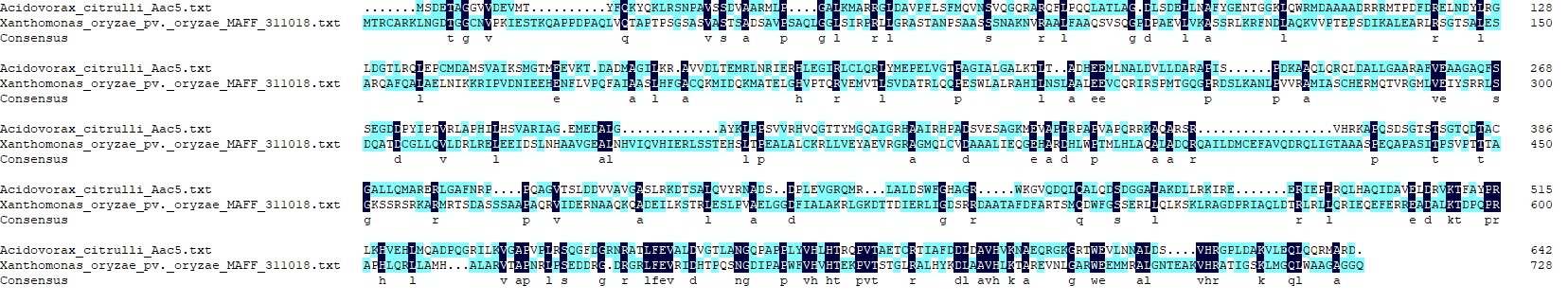

Supplement: Supplementary Figure 1 — AopP homology analysis. Analysis of amino acid homology of AopP with XopP (Xoo3222) (Ishikawa et al., 2014). [file Image_1.JPEG]

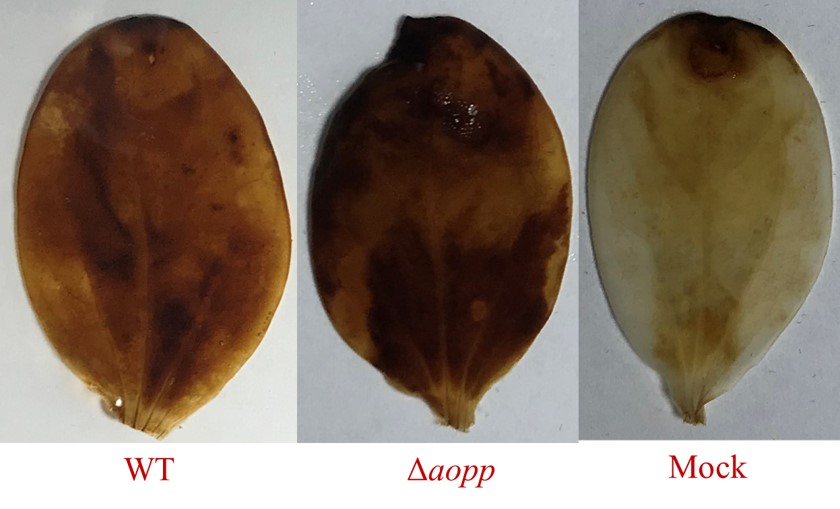

Supplement: Supplementary Figure 2 — DAB staining for detecting ROS with WT and the AopP mutant in watermelon. DAB staining analysis of ROS burst in watermelon leaves. The WT Aac5 strain and AopP mutant were cultivated to a logarithmic phase, resuspended with 10 mM MgCl2 at 1 × 10^8^ CFU/mL, and then injected into the watermelon leaves. After 24 h, the leaves were stained with DAB solution. After staining, images were captured with a camera. [file Image_2.JPEG]

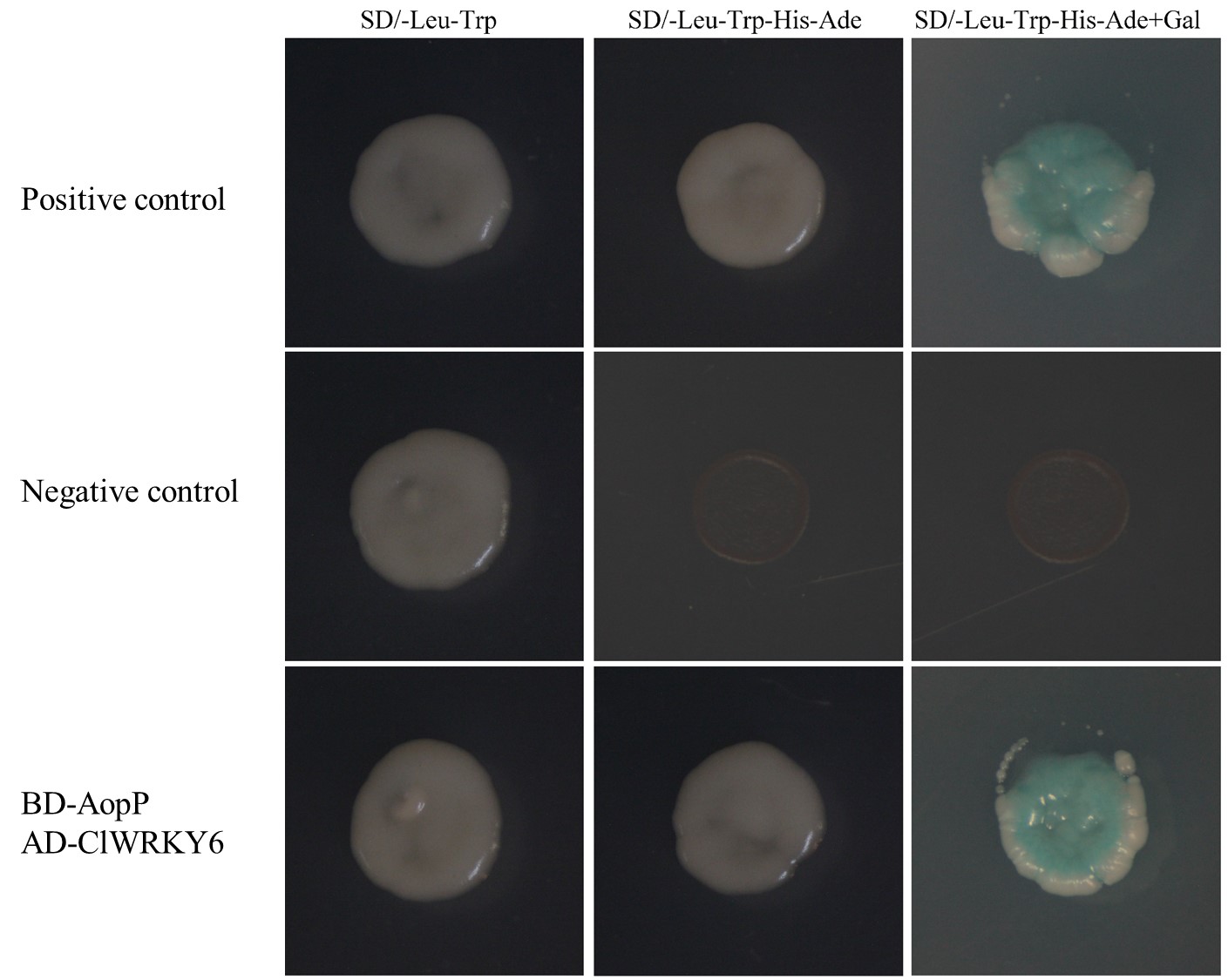

Supplement: Supplementary Figure 3 — Yeast two-hybrid with AopP and ClWRKY6. Cloning the AopP and ClWRKY6 coding sequences inserted into pGBKT7 and pGADT7 vectors, respectively. Subsequent co-transformation of the Gold2 strain and set positive control (co-transformation of pGBKT7-53 and pGADT7-T into Gold2 strain) and negative control (co-transformation of pGBKT7-lam and pGADT7-T into Gold2 strain). The Gold2 yeast strain was transformed with the above constructs, and the yeast assay was performed as described previously (Yang et al., 2014). Each experiment was independently repeated three times. [file Image_3.JPEG]

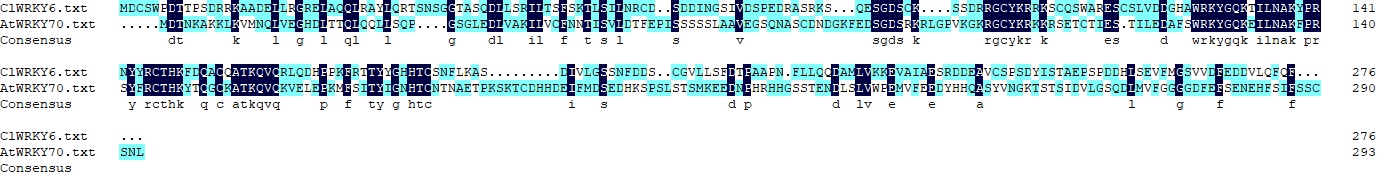

Supplement: Supplementary Figure 4 — ClWRKY6 homology analysis. Analysis of amino acid homology of ClWRKY6 with WRKY70 (Uniport: Q9LY00). The ClWRKY6 coding sequence is gene ID Cla97C10G206240 according to 97103 Watermelon V2 genome. [file Image_4.JPEG]
