# Supplementary material for: Acidovorax citrulli Type III Effector AopP Suppresses Plant Immunity by Targeting the Watermelon Transcription Factor WRKY6
Source: Front Plant Sci. 2020 Nov 20;11:579218. doi: 10.3389/fpls.2020.579218 (PMC7718035; doi:10.3389/fpls.2020.579218)
Supplement: Supplementary Table 1 — Bacterial strains and plasmids used in this study. [file Table_1.DOCX]

| **Table S1 Bacterial strains and plasmids used in this study** | | |
| --- | --- | --- |
| **Strains and plasmids** | **Characteristics^a^** | **Reference** |
| *Acidovorax citrulli* strains |  |  |
| Aac5 | Wild-type group II strain; Amp^R^ | Yan et al., 2013 |
| Δ*aopP* | *aopP* markerless mutation of Aac5; Amp^R^ | This study |
| Δ*hrcJ* | *hrcJ* mutation of Aac5; Amp^R^ | This study |
| WT-*aopP*-cyaA | Wild-type strain Aac5 containing pBBRNolac-cyaA carrying *aopN* with its native promoter; Amp^R^; Km^R^ | This study |
| Δ*hrcJ*-*aopP*-cyaA | Δ*hrcJ* containing pBBRNolac-cyaA carrying *aopN* with its native promoter; Amp^R^; Km^R^ | This study |
| D36E | The derivative strain of *Pst* DC3000, with 36 effectors deleted | Wei et al., 2018 |
| D36E-AopP-4×FLAG | D36E containing pBBRavrPto1-4×FLAG carrying AopP from 51-642 amino acid with avrPto1 promoter and its T3SS secretion signal peptide; Km^R^ | This study |
| D36E-EV | D36E containing pBBRavrPto1-4×FLAG vector with avrPto1 promoter and its T3SS secretion signal peptide; Km^R^ | This study |
| *Escherichia coli* |  |  |
| *DH5α* | *supE44* *ΔlacU169* (*Φ80/lacZ*ΔM15)  *hsdR17 recA1 endA1 gyrA96*  *thi-1 relA1* | TIANGEN |
| Plasmids |  |  |
| pK18mobsacB | Suicide vector with *sacB* gene; Km^R^ | Kvitko and Collmer, 2011 |
| pK18aopP | Suicide vector containing upstream and downstream fragments of *aopP* gene on pK18mobsacB; Km^R^ | This study |
| pBBRNolac-4FLAG | *lac* promoter was deleted from pBBR1MCS-2 and C-terminal 4×FLAG tag was inserted; need native promoter to drive only. | Zhang et al., 2018 |
| pBBRNolac-cyaA | pBBRNolac-4FLAG containing *cyaA* gene without promoter | This study |
| pBBRNolac-aopP-cyaA | pBBRNolac-cyaA containing *aopP* gene with its native promoter | This study |
| pBBRavrPto1-4×FLAG | The avrPto1 native promoter and its T3SS secretion signal peptide was introduced into pBBRNolac-4FLAG | This study |
| pBBRavrPto1-cyaA | pBBRavrPto1-4×FLAG containing *cyaA* gene | This study |
| pBBRavrPto1-AopP-4×FLAG | pBBRavrPto1-4×FLAG fused AopP from 51-642 amino acids | This study |
| pBBRavrPto1-AopP-cyaA | pBBRavrPto1-cyaA fused AopP from 51-642 amino acids |  |
| pBI121 | plant expression vector containing 35S promoter, Km^R^ | This study |
| pBI121-3×FLAG | pBI121 fused 3×FLAG tag | This study |
| pBI121-GFP | pBI121 fused eGFP tag | This study |
| pBI121-3×FLAG-AopP | pBI121-3×FLAG fused AopP | This study |
| pBI121-GFP-AopP | pBI121-GFP fused AopP | This study |
| pBI121-mCherry | pBI121 fused mCherry tag | This study |
| pBI121-mCherry-ClWRKY6 | pBI121-mCherry fused ClWRKY6 | This study |
| pCAMBIA1300-nLUC | plant expression vector containing 35S promoter, carrying nLUC tag, Km^R^ | Chen et al., 2008 |
| pCAMBIA1300-cLUC | plant expression vector containing 35S promoter, carrying cLUC tag, Km^R^ | Chen et al., 2008 |
| pCAMBIA1300-nLUC-AopP | pCAMBIA 1300-cLUC fused AopP | This study |
| pCAMBIA1300-cLUC-ClWRKY6 | pCAMBIA1300-cLUC fused ClWRKY6 | This study |
| pSPYNE®173 | plant expression vector containing 35S promoter, carrrying nYFP tag, Km^R^ | Waadt et al., 2008 |
| pSPYCE(M) | plant expression vector containing 35S promoter, carrrying cYFP tag, Km^R^ | Waadt et al., 2008 |
| pSPYNE®173-AopP | pSPYNE®173 fused AopP | Lab collection |
| pSPYCE(M)-ClWRKY6 | pSPYCE(M) fused ClWRKY6 | Lab collection |
| pET22b(+) | Prokaryotic expression vector, carrying His_6_ tag, Amp^R^ | Lab collection |
| pGEX6P-1 | Prokaryotic expression vector, carrying GST tag, Amp^R^ | Lab collection |
| pET22b(+)-ClWRKY6 | pET22b(+) fused ClWRKY6 | This study |
| pGEX6P-AopP | pGEX6P fused AopP | This study |
| pRK600 | Helper strain in tri-parental mating; Cm^R^ | Lab collection |
| ^a^Amp^R^, Km^R^, and Rif^R^ , and Cm^R^ indicate resistance to ampicillin, kanamycin, rifampicin, and chloramphenicol, respectively | | |
