# Supplementary material for: Acidovorax citrulli Type III Effector AopP Suppresses Plant Immunity by Targeting the Watermelon Transcription Factor WRKY6
Source: Front Plant Sci. 2020 Nov 20;11:579218. doi: 10.3389/fpls.2020.579218 (PMC7718035; doi:10.3389/fpls.2020.579218)
Supplement: Supplementary Table 2 — List of all the primer sequences used in this study. [file Table_2.docx]

|  | | **Table S2. Primers used in this study** | | |
| --- | --- | --- | --- | --- |
| **Primer name** | **Sequence (forward/reverse)** | | **Length** | **Description** |
| aopP-1F | CTATGACATGATTACGAATTCGGTCCCATCGTCAGGCGGTCTA | | 285 bp | For deleting the *aopP* gene, located upstream of the *aopP* gene |
| aopP-1R | CGTGGCGGCATCAGTCCCGCCCGCTGTCTCATCGCT | |  |  |
| aopP-2F | AGCGATGAGACAGCGGGCGGGACTGATGCCGCCACG | | 364 bp | For deleting the *aopP* gene, located downstream of the *aopP* gene |
| aopP-2R | CAGGTCGACTCTAGAGGATCCGGCGTTCAGGCCGTGCAGTT | |  |  |
| aopP-TF | GCCGTGCCATTCCTGAGCT | | 337 bp | For confirming the deletion of the *aopP* mutant |
| aopP-TR | CCATGTCCGCGTCGGTTTT | |  |  |
| HBaopN-F | CGCTCTAGAACTAGTGGATCCGCGTTGCGGCGAAGCGTTTC | | 2426 bp | For cloning the *aopN* full-length ORF carrying native promo ter sequence and constructed the pBBRNolac-aopP-cyaA vector |
| HBaopN-R | CGATTGCTGCCCATCGAATTCGTCCCGGGCCATGCGCTGCT | |  |  |
| Km-F | ATGATTGAACAAGATGGATTGCAC | | 795 bp | Confirming the absence of Kan resistance fragment in the deletion mutants |
| Km-R | TCAGAAGAACTCGTCAAGAAGGC | |  |  |
| WFB1 | GACCAGCCCACACTGGGAC | | 360 bp | For confirming *Acidovorax citrulli* strains |
| WFB2 | CTGCCGCACTCCAGCGA | |  |  |
| avrPto-F | CGCGGTGGCGGCCGCTCTAGAAGCCAAGGCAGCGTTACTGT | | 315 bp | For cloning the *avrPto* native promoter and its its T3SS secretion signal peptide was inserted into pBBRNolac vector, and constructed the pBBRavrPto-4×FLAG vector |
| avrPto-R | TTCCTGCAGCCCGGGGGATCCTGGTACACCAGCAGACTCCG | |  |  |
| 121GFP-aopP-F | GAGAACACGGGGGACTCTAGAATGAGCGATGAGACAGCGGG | | 1926 bp | For cloning the *aopP* full-length ORF introducing pBI121-GFP vector |
| 121GFP-aopP-R | GCCCTTGCTCACCATCCCGGGGTCCCGGGCCATGCGCT | |  |  |
| Ac-cyaA-F | TCCCCCGGGCTGCAGGAATTC GATGGGCAGCAATCGCATCA | | 1221 bp | For cloning the *cyaA* introducing pBBRNolac vector, and constructed the pBBRNolac-cyaA vector |
| Ac-cyaA-R | ATCCTTGTAATCGGTAAGCTT GCTGTCATAGCCGGAATCCT | |  |  |
| D36E-cyaA-F | TCCCCCGGGCTGCAGGAATTC GATGGGCAGCAATCGCATCA | | 1221 bp | For cloning the *cyaA* introducing pBBRavrPto-4×FLAG vector, and constructed the pBBRavrPto-cyaA vector |
| D36E-cyaA-R | ATCCTTGTAATCGGTAAGCTT GCTGTCATAGCCGGAATCCT | |  |  |
| D36E-aopP-F | TCCCCCGGGCTGCAGGAATTCGCCGTGCCATTCCTGAGCTT | | 1776 bp | Cloning starts with 51 amino acids of AopP introducing pBBRavrPto-4×FLAG vector, and constructed the pBBRavrPto-AopP-4×FLAG vector |
| D36E-aopP-R | GGTAAGCTTGATATCGAATTCGTCCCGGGCCATGCGCTG | |  |  |
| D36E-cyaA-aopP-F | TCCCCCGGGCTGCAGGAATTC GCCGTGCCATTCCTGAGCTT | | 1776 bp | Cloning starts with 51 amino acids of AopP introducing pBBRavrPto-cyaA vector, and constructed the pBBRavrPto-AopP-cyaA vector |
| D36E-cyaA-aopP-R | CGATTGCTGCCCATCGAATTCGTCCCGGGCCATGCGCTG | |  |  |
| 121FLAG-aopP-F | GAGAACACGGGGGACTCTAGAATGAGCGATGAGACAGCGGG | | 1926 bp | For cloning the *aopP* full-length ORF introducing pBI121-3×FLAG vector |
| 121FLAG-aopP-R | GTCATCCTTGTAATCCCCGGGGTCCCGGGCCATGCGCT | |  |  |
| 121FLAG-F | GGACTCTAGAGGATCCCCGGGGATTACAAGGATGAC | | 75 bp | For inserting the 3×FLAG tag into pBI121 vector, and constructed the pBI121-3×FLAG vector |
| 121-FLAG-R | CGATCGGGGAAATTCGAGCTCTCACTTGTCATCGTCAT | |  |  |
| 121GFP-F | GGACTCTAGAGGATCCCCGGGATGGTGAGCAAGGGCGAG | | 720 bp | For inserting the eGFP tag into pBI121 vector, and constructed the pBI121-GFP vector |
| 121GFP-R | CGATCGGGGAAATTCGAGCTCTCACTTGTACAGCTCGTCCATG | |  |  |
| mCherry-F | GGACTCTAGAGGATCCCCGGGATGGTGAGCAAGGGCGAGGA | | 711 bp | For inserting the mCherry tag into pBI121 vector, and constructed the pBI121-mCherry vector |
| mCherry-R | CGATCGGGGAAATTCGAGCTCTCACTTGTACAGCTCGTCCATG | |  |  |
| mCherry-WRKY6-F | GAGAACACGGGGGACTCTAGAATGGATTGTTCCTGGCCTG | | 828 bp | For cloning the *ClWRKY6* full-length ORF introducing pBI121-mCherry vector |
| mCherry-WRKY6-R | GCCCTTGCTCACCATCCCGGGAAACTGGAATTGTAAAACATCAT | |  |  |
| 22b-WRKY6-F | TAAGAAGGAGATATACATATGGATTGTTCCTGGCCTGACA | | 825 bp | For cloning the *ClWRKY6* introducing pET22b(+) vector |
| 22b-WRKY6-R | GTGGTGGTGGTGGTGCTCGAGAAACTGGAATTGTAAAACATC | |  |  |
| 6P-AopP-F | TTCCAGGGGCCCCTGGGATCCAGCGATGAGACAGCGGGC | | 1926 bp | For cloning the *aopP* introducing pGEX6P-1 vector |
| 6P-AopP-R | GTCACGATGCGGCCGCTCGAGTCAGTCCCGGGCCATG | |  |  |
| nLUC-aopP-F | ACGGGGGACGAGCTCGGTACCATGGAACAAAAGCTAATCTCCGAGGAAGACTTGAGCGATGAGACAGCGGG | | 1923 bp | For cloning the *aopP* introducing pCAMBIA1300-nLUC vector |
| nLUC-aopP-R | CGCGTACGAGATCTGGTCGACGTCCCGGGCCATGCGCT | |  |  |
| cLUC-WRKY6-F | TACGCGTCCCGGGGCGGTACCATGGATTGTTCCTGGCCTG | | 828 bp | For cloning the *ClWRKY6* full-length ORF introducing pCAMBIA1300-cLUC vector vector |
| cLUC-WRKY6-R | ACGAAAGCTCTGCAGGTCGACTCAAGCGTAATCTGGAACATCGTATGGGTAAAACTGGAATTGTAAAACATCAT | |  |  |
| 173-aopP-F | CCCAGGCCTACTAGTGGATCCAGCGATGAGACAGCGGGC | | 1926 bp | For cloning the *aopP* introducing pSPYNE®173 vector |
| 173-aopP-R | CCCGGGAGCGGTACCCTCGAGTCAGTCCCGGGCCATG | |  |  |
| M-WRKY6-F | TGGCGCGCCACTAGTGGATCCATGGATTGTTCCTGGCCTG | | 828 bp | For cloning the *ClWRKY6* introducing pSPYCE(M) vector vector |
| M-WRKY6-R | CCCGGGAGCGGTACCCTCGAGAGCGTAATCTGGAACATCGTATGGGTAAAACTGGAATTGTAAAACATCAT | |  |  |
| NbPti5-F | CCTCCAAGTTTGAGCTCGGATAGT | |  | For detecting *NbPti5* mRNA in q-PCR assay; Chen et al., 2015 |
| NbPti5-R | CCAAGAAATTCTCCATGCACTCTGTC | |  |  |
| NbAcre31-F | AATTCGGCCATCGTGATCTTGGTC | |  | For detecting *NbAcre31* mRNA in q-PCR assay; Chen et al., 2015 |
| NbAcre31-R | GAGAAACTGGGATTGCCTGAAGGA | |  |  |
| NbGras2-F | TACCTAGCACCAAGCAGATGCAGA | |  | For detecting *NbGras2* mRNA in q-PCR assay; Chen et al., 2015 |
| NbGras2-R | TCATGAGGCGTTACTCGGAGCATT | |  |  |
| NbEF1α-F | AAGGTCCAGTATGCCTGGGTGCTTGAC | |  | Reference gene of *N.benthamiana* in q-PCR assay; Chen et al., 2015 |
| NbEF1α-R | AAGAATTCACAGGGAC AGTTCCAATACCA | |  |  |
| RT-aopP-F | CCGGATTTCGATCGGGAACT | | 125 bp | For detecting *aopP* mRNA in q-PCR assay |
| RT-aopP-R | TTGACTTCCTCCATCGTGCC | |  |  |
| RT-WRKY6-F | GAAGAAAGAGTTGCCAGAGT | | 137 bp | For detecting *ClWRKY6* mRNA in q-PCR assay |
| RT-WRKY6-R | GAATTTGTGGGTGCATCTGT | |  |  |
| WACTIN | CCATGTATGTTGCCATCCAG | |  | Reference gene of Watermelon in q-PCR assay; Kong et al., 2014 |
| WACTIN | GGATAGCATGGGGTAGAGCA | |  |  |
| RT-rpoB-F | GCGACAGCGTGCTCAAAGTG | | 104 bp | Reference gene of *A.citrulli* strain Aac5 in q-PCR assay; Zhang et al., 2018 |
| RT-rpoB-F | GCCTTCGTTGGTGCGTTTCT | |  |  |
